# Pure and multi metal oxide nanoparticles: synthesis, antibacterial and cytotoxic properties

**DOI:** 10.1186/s12951-016-0225-6

**Published:** 2016-10-24

**Authors:** Slavica Stankic, Sneha Suman, Francia Haque, Jasmina Vidic

**Affiliations:** 1 CNRS, Institut des Nanosciences de Paris (INSP), UMR 7588, 4 Place Jussieu, 75252 Paris Cedex 05, France; 2 UPMC-Université Paris 06, INSP, UMR 7588, Paris, France; 3 Birla Institute of Technology & Science, Pilani Campus, Vidya Vihar, Pilani, Rajasthan India; 4 Virologie et Immunologie Moléculaires, UR892, INRA, Paris Saclay University, Jouy en Josas, France; 5 School of Material Science and Engineering, Nanyang Technological University, 50 Nanyang Ave, Singapore, 639798 Singapore; 6 NTU-HJU-BGU CREATE Programme, 1 Create Way, Research Wing # 02-06 to 08, Singapore, 138602 Singapore

**Keywords:** Multi-metal oxide nanoparticles, Nanoparticles synthesis, Antibacterial activity, Cytotoxicity

## Abstract

Th antibacterial activity of metal oxide nanoparticles has received marked global attention as they can be specifically synthesized to exhibit significant toxicity to bacteria. The importance of their application as antibacterial agents is evident keeping in mind the limited range and effectiveness of antibiotics, on one hand, and the plethora of metal oxides, on the other, along with the propensity of nanoparticles to induce resistance being much lower than that of antibiotics. Effective inhibition against a wide range of bacteria is well known for several nano oxides consisting of one metal (Fe_3_O_4_, TiO_2_, CuO, ZnO), whereas, research in the field of multi-metal oxides still demands extensive exploration. This is understandable given that the relationship between physicochemical properties and biological activity seems to be complex and difficult to generalize even for metal oxide nanoparticles consisting of only one metal component. Also, despite the broad scope that metal oxide nanoparticles have as antibacterial agents, there arise problems in practical applications taking into account the cytotoxic effects. In this respect, the consideration of polymetallic oxides for biological applications becomes even greater since these can provide synergetic effects and unify the best physicochemical properties of their components. For instance, strong antibacterial efficiency specific of one metal oxide can be complemented by non-cytotoxicity of another. This review presents the main methods and technological advances in fabrication of nanostructured metal oxides with a particular emphasis to multi-metal oxide nanoparticles, their antibacterial effects and cytotoxicity.

## Review

### Background

Nanomaterials have numerous applications in areas ranging from catalysis, photonics, molecular computing, energy storage, fuel cells, tunable resonant devices, sensing to nanomedicine. This is due to an increase in reactivity when compared to their micro-sized counterparts since nanoscaled materials exhibit larger surface-to-volume ratio which provides unsaturated and, thus, more reactive surface atoms. To consider nanoparticles for biological applications, such as drug delivery, biosensing, imaging and antibacterial therapeutics, several key requirements have to be fulfilled. The first is to deal with the engineered nanoparticles of well characterized composition, size, crystallinity and morphology. The second implies manipulation of stabilized, non-agglomerated nanomaterials in order to control dosing. Finally, the most crucial requirement is their biocompatibility. Despite very fast expansion of the bionanotechnology in the last 30 years, there are many challenges facing these three requirements. Relevant works that aimed at correlating synthesis, stabilization and surface modification of nanoparticles with their biological effects and decreased toxicity have shown that there is no general rule.

Presently, microbial resistance to antibiotics has been reaching a critical level. In exploring various options to address this problem, inorganic nanomaterials, like metal oxide nanoparticles, have emerged as promising candidates since they possess greater durability, lower toxicity and higher stability and selectivity when compared to organic ones. Nanostructured metal oxides have already been extensively studied for their promising use in technology. This has resulted in development of numerous reproducible procedures for the synthesis of nanoparticles with desired characteristics—like size, shape, morphology, defects in the crystal structure, monodispersity—providing a rich background for research relevant to antibacterial applications. Characterization of these nanoparticles can be helpful in modifying and tuning their antibacterial and cytotoxic effects. For instance, it has been established that the antibacterial activity increases with decreasing the particles size [1]. In contrast, the crystallographic orientation appears to have no effect on antibacterial activity [2], whereas increasing the lattice constants enhances the antibacterial activity [3]. It has also been proposed that different morphologies and crystal growth habits can affect the antibacterial activity [4]. Hence, the synthesis technique employed is functional in determining the biological characteristics of a given nanoparticle. As potential novel antibacterial agents, metal oxide nanoparticles like Fe_3_O_4_, TiO_2_, CuO and ZnO are being thoroughly investigated. Their relatively low toxicity against human cells [5], low cost [6], size-dependent effective inhibition against a wide range of bacteria, ability to prevent biofilm formation [7] and even eliminate spores [8] make them suitable for application as anti-bacterial agents in the fabric [7], skincare products [9], biomedical [10] and food-additive industries [11]. However, research to understand cytotoxic effects and the corresponding mechanisms is necessary to adapt this class of nanomaterials for safe applications.

Recent achievements in nanotechnology of metal oxides include elaboration of nanostructured oxides consisting of two or more metallic components. Their potential applications are immense due to their unique electronic, optical, magnetic and other physicochemical properties [12]. Multi-metal oxide nanoparticles, like Zn_x_Mg_1−x_O, Ta-doped ZnO, Ag/Fe_3_O_4_ nanocomposites, are being studied extensively as potential antimicrobial agents owing to the beneficial synergistic effects of their components. These nanoparticles have shown promising solutions to problems seen in pure metal oxide nanoparticles, like high cytotoxicity or agglomeration. In this paper, we have discussed the existing synthesis routes and the antibacterial activity of metal oxide nanoparticles with a particular focus on polymetallic oxides. Additionally, a strong emphasis has been given to their cytotoxic nature.

### Synthesis methods of metal oxide nanoparticles

**Fig. 1:**
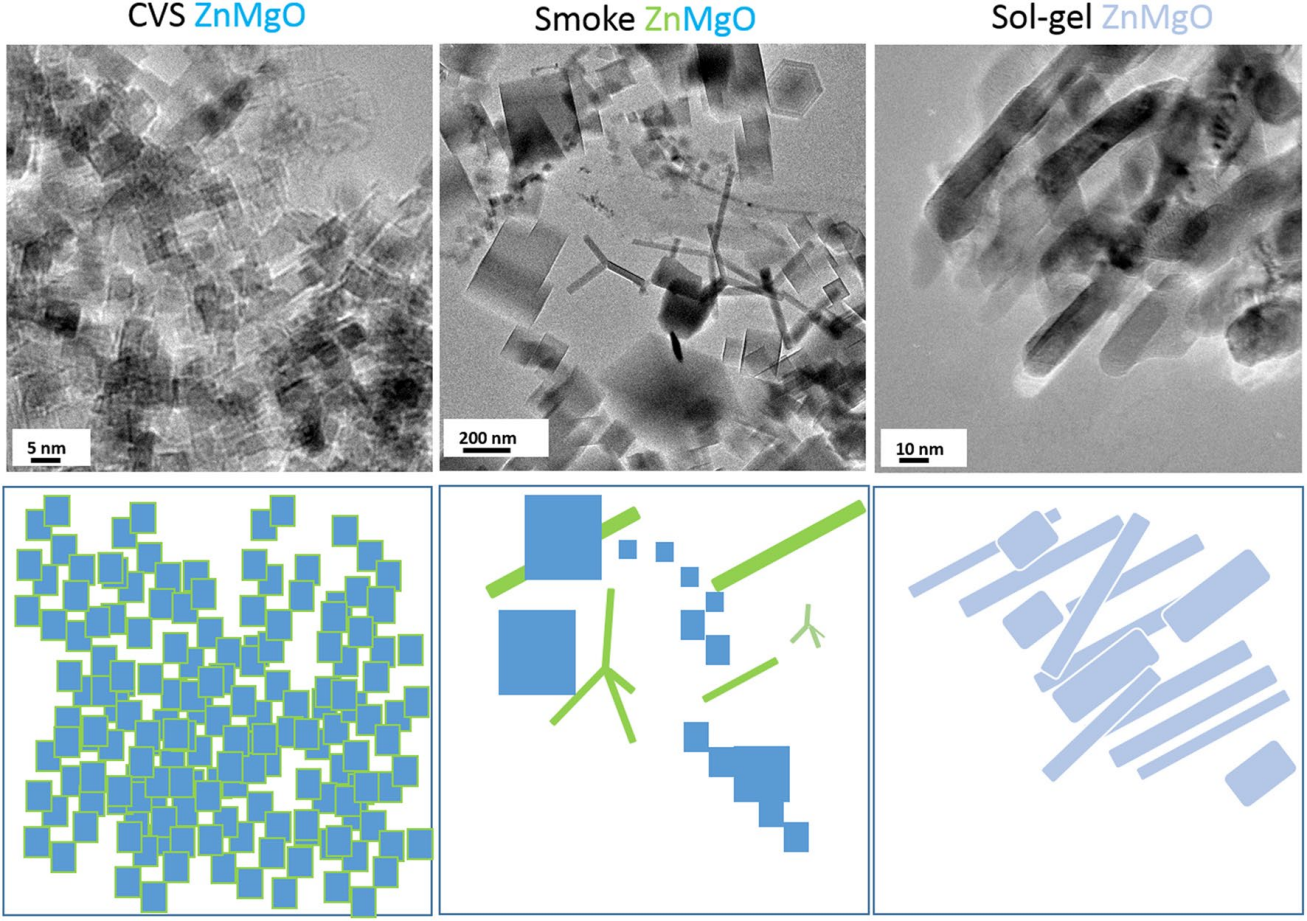
Various shapes of ZnMgO nanoparticles produced by dufferent synthesis routes. TEM images of ZnMgO nanoparticles obtained via three different synthesis methods at the Paris Institute of Nanosciences and the illustrations of the corresponding crystal forms. All powders were kept at P < 10^−5^ mbar after the synthesis while the microscopic measurements were performed on bare powders in order to analyze the initial morphology resulting from the corresponding fabrication route. Surface segregation of Zn-atoms is highlighted by green color surrounding MgO cubes in the illustration representing CVS method

Before exploring the antibacterial properties of metal oxide nanoparticles, a review of the various synthesis methods has been described. We make broadly a division of synthesis methods into three categories: solution based, vapor state and biological methods. Such division is based on the type of the medium in which the oxidation reaction takes place. The choice of synthesis method determines the physicochemical characteristics of the metal oxide nanoparticle, such as the size, dispersity, type of intrinsic and/or extrinsic defects, morphology and crystal structure. An example is given in Fig. 1 for nano-ZnMgO fabricated via three different synthesis methods. Corresponding TEM images show that this polymetallic oxide can be found in form of regular cubes of similar size (chemical vapor synthesis, CVS ZnMgO), a mixture of cubes and tetrapods (metal combustion, Smoke ZnMgO) and irregular nanorods (sol–gel ZnMgO). It was, furthermore, shown that despite cubic and hexagonal phase, that are thermodynamically most stable for pure MgO and ZnO, respectively, CVS technique allows for stabilization of one crystal structure in ZnMgO. Diffractions specific of only cubic crystal phase were observed in the corresponding XRD pattern while other measurements demonstrated that Zn-atoms replace Mg atoms on the surface of nanocubes [13]. The surface segregation of Zn-atoms is highlighted by green color surrounding cubes in the illustration of Fig. 1. In contrast, phase separation is most probably the reason for the presence of two types of shapes in ZnMgO powder obtained via metal combustion.

All these physicochemical properties, that are evidently in a strong correlation with the synthesis route, determine nanoparticles surface energies and, thus, their interaction with biological entities.

#### Solution based synthesis

##### Sonochemical method

In sonochemical methods, solution of the starting material (for e.g. metallic salts) is subjected to a stream of intensified ultrasonic vibrations which breaks the chemical bonds of the compounds. The ultrasound waves pass through the solution causing alternate compression and relaxation. This leads to acoustic cavitation i.e. formation, growth and implosive collapse of bubbles in the liquid. In addition, the change in pressure creates microscopic bubbles that implode violently leading to emergence of shock waves within the gas phase of the collapsing bubbles. Cumulatively, the effect of millions of bubbles collapsing produces an excessive amount of energy that is released in the solution. Transient temperatures of ~5000 K, pressure of ~1800 atm and cooling rates above 10^10^ K/s have been recorded at the localized cavitational implosion hotspots [14]. The excessively high rate of cooling process is found to affect the formation and crystallization of the obtained products [15]. This method has been used to synthesize a wide range of nanomaterials as metals, alloys, metal oxides, metal sulfides, metal nitrides, metalpolymer composites, metal chalcogenides, metal carbides etc. [16]. Examples of reported metal oxides synthesized by this method include TiO_2_ [17], ZnO [18], CeO_2_ [19], MoO_3_ [20], V_2_O_5_ [21], In_2_O_3_ and Eu/Dy-doped In_2_O_3_ [22], ZnFe_2_O_4_ [23], PbWO_4_ [24], BiPO_4_ [25], ZnAl_2_O_4_ and ZnGa_2_O_4_—pure and doped with varying combinations of Dy^+3^, Tb^+3^, Eu^+3^ and Mn^+2^ [26], Fe_3_O_4_ [27], BaFe_12_O_19_ [28] and Mn-doped *γ*-Fe_2_O_3_ [29]. Using this method, enhanced photocatalytic properties in the case of TiO_2_ [17] or varying magnetism of iron-oxide nanoparticles [30–32] have been reported. The advantages associated with sonochemical methods include uniform size distribution, a higher surface area, faster reaction time and improved phase purity of the metal oxide nanoparticles as observed by various research groups mentioned in references listed above.

##### Co-precipitation method

**Co-precipitation meth**od involves precipitating the oxo-hydroxide form from a solution of a salt precursor (metal salts like nitrates or chlorides) in a solvent (like water) by using a precipitating medium. Once a critical concentration of species in solution is reached, a short burst of nucleation occurs followed by growth phase. This method has been employed in synthesizing metal oxides like ZnO [33], MnO_2_ [34], BiVO_4_ [35], MgO [36], Ni_1-x_Zn_x_Fe_2_ O_4_  [37], SnO_2_ [38], Cu-doped ZnO [39], MgFe_2_O_4_ [40], Ni–CeZrO_2_ [41] and Y_2_O_3_:Eu^+3^ [42]. Co-precipitation is commonly used for preparing magnetic nanoparticles such as magnetite by using a base, usually NaOH and NH_4_OH, for alkaline co-precipitation of ferrous and ferric salts dissolved in water in stoichiometric amounts [34, 43, 44]. The use of NaOH, KOH and (C_2_H_5_)_4_NOH as a precipitating medium has established that pH, the nature of alkali, the slow or fast addition of alkaline solution and the drying modality of synthesized powders affect the size, paramagnetic properties and degree of agglomeration of the synthesized magnetite nanoparticles [44]. In addition, the use of surfactants has been seen to be useful in optimizing further the surface characteristics [42]. The advantages of this method are low cost, mild reaction conditions like low synthesis temperature, the possibility to perform direct synthesis in water, simplicity of processing, the ease of scale-up, flexibility in modulation of core and surface properties [39, 44].

##### Solvothermal method

These methods are employed to prepare a variety of nanomaterials by dispersing the starting material in a suitable solvent and subjecting it to moderately high temperature and pressure conditions which lead to product formation. An organometallic complex of titanium, orthobutoxide, was for instance used for the synthesis of TiO_2_ nanoparticles [45]. When the reaction is performed using water as the solvent, the method is called hydrothermal synthesis. Chemical parameters (type, composition and concentration of the reactants, ratio-solvent/reducing agent) and thermodynamic parameters (temperature, pressure and reaction time) affect the final particle formation. It was also observed that basicity and hydrolysis ratio of the reacting medium together with the steric or electrostatic stabilization of the reactive molecules affect the nucleation and growth steps, which in turn control the particle size, shape, composition and crystal structure of particles. For instance, varying the hydrolysis ratio allows to synthesize either metal or (oxy)hydroxide or oxide nanoparticles [46]. Nanoparticles of Nb_2_O_5_, MgO, TiO_2_, MnFe_2_O_4_, CoFe_2_O_4_ and Fe_3_O_4_ have been synthesized using polyol as the solvent [46–50]. Solvothermal methods have successfully been employed to prepare various nanocomposites displaying a combination of the properties of their parent nanoparticles. Zhai et al. [51] have synthesized novel water-soluble nanohybrids composed of shape-tuned Ag cores and a Fe_3_O_4_ shell. Graphene-TiO_2_ nanocomposites [52], CoFe_2_O_4_@BaTiO_3_ nanocomposites [53], a series of multifunctional magnetic core–shell hetero-nanostructures (Fe_3_O_4_@NiO and Fe_3_O_4_@Co_3_O_4_) [54] are some other examples. This method, moreover, allows for the preparation of ultra-small nanoparticles (<5 nm) such as 2.5 × 4.3 nm TiO_2_ nanoparticles [55] and 1.6 ± 0.3 nm WO_x_ nanoparticles [56]. In the latter case, it was shown that, the use of reducing/oxidizing agents may strongly affect both, the size (use of an oxidizing agent led to particles with diameters smaller then 1 nm) and the shape (use of a reducing agent led to rod-shaped nanoparticles). Tian et al. have shown that adjusting the ratio of reducing agent and solvent can tune the particle size of magnetite nanoparticles from ~6 to 1 nm [57] while iron oxide nanostructures could be produced in different morphologies—such as, nanocubes [58] and hollow spheres [59]—by this synthesis route. Another advantage of this technique is the use of suitable surfactants that can tune the particle characteristics and limit their agglomeration. For example, using a zwitterionic surfactant, smaller ZnO particle sizes were obtained as compared with those obtained from surfactant-free hydrothermal reaction [60]. Du et al. have reported surfactant assisted solvothermal technique to prepare mixed metal oxide nanoparticles like barium ferrite (BaFe_12_O_19_) and Co-Ti-doped barium-ferrite nanoparticles (Ba(CoTi) × Fe_12_ – 2 × O_19_) with high-purity crystalline phase, small particle size and good magnetic properties [61].

##### Sol–gel method

Main steps of sol–gel method include the hydrolysis of metalorganic compound precursors, like alcoxysilane [62] to produce corresponding oxo-hydroxide, followed by condensation to form a network of the metal hydroxide. After hydroxide polymerizes it forms a dense porous gel the subsequent drying and heating of which leads to the production of ultrafine porous oxides in the desired crystal phase. The method has been used to synthesize a variety of metal oxide nanoparticles, like TiO_2_ [63], ZnO [64], MgO [65], CuO [66], ZrO_2_ and Nb_2_O_5_ [67] and nanocomposites, like LiCoO_2_ thin film [68], Cu doped ZnO nanoparticles [69], CuO/Cu_2_O nanocomposites [70], Ce-doped ZrO_2_ [71], oxides of Hf, Ta and Nb [72]. Moreover, sol gel method is promising in doping of Group 5 oxides, which is generally a challenge. It is seen as a clean, surfactant free technique to synthesize high quality nanocrystals of doped metal oxide nanoparticles with magnetic properties like cobalt doped Hf-oxide nanoparticles [72]. To eliminate/reduce limitations associated with this method, researchers have incorporated certain modifications. For instance, Corr et al. have reported a modified one-step sol–gel aqueous approach for the synthesis of iron oxide-silica nanocomposite [62]. The modification consisted of employing ultrasonic conditions to overcome the effects of high temperature conditions (up to 600 °C) which could lead to oxidation of the products. Under the effect of ultrasound vibrations, high temperatures and pressures could instantaneously be generated and then dissipated in the local environment of the particles avoiding oxidation [73]. This technique has also been used to prepare novel nanocomposites like InNbO_4_, a photocatalytically active ternary metal oxides semiconductor [74]. Sol–gel method, moreover, allows for a formation of multi-metal oxides instead of a mixture of the individual binary oxides—as shown for SnO_2_-doped In_2_O_3_ [75]. Also it provides the particle size to be tuned by simply varying the gelation time [76]. In addition, it has been reported that supercritical fluids can be used to synthesize nanoparticles like TiO_2_, ZrO_2_, Al_2_O_3_, TiO_2_-SiO_2_, SiO_2_-Al_2_O_3_ and ZrO_2_/TiO_2_ hybrid oxide nanotubes [77].

##### Microwave-assisted method

This method has been of increasing interest as it is relatively low energy and time consuming [78]. The reaction times are reduced from a few hours to several minutes without compromising the particle purity or size. Faster reaction rates can be achieved by employing high heating rates which favor rapid nucleation and formation of small, highly monodisperse particles. Microwave-assisted methods involve quick and uniform heating of the reaction medium with no temperature gradients through two mechanisms: dipolar polarization and ionic conduction. Highly crystalline nanoparticles of MnO, Fe_3_O_4_, CeO_2_, CaO, BaTiO_3_, ZnO, Cr_2_O_3_, CoO, Mn_2_O_3_ and MgO have been successfully synthesized using microwave-assisted routes [79–82]. Automation allows control over the reaction conditions and hence facilitates manipulation of particle size, morphology and crystallinity [83]. The choice of starting metal oxides precursors (as acetates, chlorides, isopropyls) and solvents (as ethylene glycol, benzene) can govern reaction success, particle size and crystal structure [80].

##### Microemulsion method

This method comprises two immiscible phases (oil and water) which are separated by a monolayer of surfactant molecules forming two binary systems—water/surfactant and oil/surfactant—such that the hydrophobic tails of the surfactant molecules are dissolved in the oil phase and the hydrophilic head groups in the aqueous phase. Broadly the method comprises of mixing appropriate amounts of the surfactant, oil, water and the metallic precursor (for instance, organometallic precursor can be added as a solution in the oily phase) by stirring at room temperature to prepare a homogenized phase [84]. Reducing/oxidizing/precipitating agents are then added, under vigorous stirring, to enable sedimentation of the nanoparticles. The microemulsions act as nanoreactors for synthesis of the nanoparticles. This is then followed by centrifugation, wash cycles and drying/calcination. Shape and size can be manipulated in these methods by affecting the various self-assembled structures formed in the binary systems [85]. This method was used to synthesize iron oxide nanoparticles [86], NiO [85], CeO_2_ [84], TiO_2_ [87], ZnO [88], CuO [89], and nanocomposites like BaAlO_2_ [90], iron-oxide doped alumina nanoparticles [91]. The ability to control the formation of different kinds of core–shell structures with sub-nanometric resolution is seen as a major benefit of this technique [92]. Additionally, the method also provides the possibility to manipulate size and morphology of nanoparticles by adjusting parameters such as concentration and type of surfactant, the type of continuous phase, the concentration of precursors and molar ratio of water to surfactant. The disadvantage associated with this method involves the necessity of several washing processes and further stabilization treatment due to aggregation of the produced nanoparticles [86]. Modifications have been incorporated to overcome these disadvantages. For instance, reverse microemulsion technique has been used to produce monodisperse spherical ZnO nanoparticles. The modification was that ZnO nanoparticles were not directly produced in the microemulsion but by the thermal decomposition of zinc glycerolate microemulsion product during subsequent calcination process [93]. The modified technique prevented agglomeration whereas the calcination temperature and concentration of surfactant could be varied in order to tune the particle size and morphology of the ZnO nanoparticles, respectively.

#### Vapor state synthesis

##### Laser ablation method

This method is used to generate nanoparticles by laser irradiation of immersed targets of colloidal solutions generated from bulk materials immersed in aqueous or non-aqueous solvents [94]. The method has been used to synthesize ZnO [95], NiO [96], SnO_2_ [97], ZrO_2_ [98], iron-oxide [99], Al_2_O_3_ [100] but also ternary metal oxides like Au-SnO_2_ [101], Cu/Cu_2_O [102]. The size of the nanoparticles can be controlled by manipulating two parameters: laser fluence and the nature of the liquid media [103, 104]. Indeed, the size of the nanoparticles increases with increase in thickness of the molten layer, which in turn increases with increase in laser fluence. The nature of the liquid plays an important role as the vapor pressure of the liquid and provides the recoil pressure under which the molten layer transforms into nanoparticles. Liu et al. [105] have established laser ablation of metal targets in aqueous environments to generate nanoparticles of oxides of Ti and Ni with well-controlled phase, size and size distribution, along with high production rate. Some of the drawbacks associated with laser ablation are related to propensity for nanoparticle agglomeration, lack of long term stabilization in solution and the need for capping [106].

##### Chemical vapor based methods

In *chemical vapor deposition* (CVD), substrates are heated to high temperatures and exposed to precursor materials in the gaseous state. The precursors react or decompose on the substrate surface to form nanomaterial. In *chemical vapor synthesis* (CVS) approach, within a flow reactor pure metal or metal–organic salts are by heating transformed into the vapor phase and introduced into a hot-wall reactor where they react with the oxidizing agent under conditions that favor the chemical [107, 108]. Usually an inert gas, such as Ar, is used to carry the gaseous reactants to the reaction zone where nucleation and crystal growth occur. Finally, the product that is also in the gas phase is carried to a much cooler zone where it due to such temperature gradient transforms into a solid state and can get collected. These techniques are extensively employed to produce uniform and contamination-free metal oxide nanoparticles and films; such as ZnO nanowires and films [109] and defect-free ZnO nanoparticles [110], nanocubes and nanospheres of magnetite [111], Cu_2_O [112], MgO and CaO [113], SnO_2_ [114], SrO [115], CoO and Co_3_O_4_ [116]. When multi-metal oxides are considered, this technique allows for the production of B-doped ZnO [117], europium doped yttria (YO: Eu) [118], Li-doped MgO [119], Ca-doped [92, 120]. Moreover, via CVS technique Zn^2+^ cations may selectively replace Mg^2+^ surface cations preferentially at the edges and corners of MgO nanocubes that resulted in unique optical and chemical surface properties of ternary Zn_x_Mg_1−x_O nanoparticles [13]. Reproducibility is another advantage associated with this method [121]. Careful choice of experimental parameters such for instance the nature and/or concentration of the oxidizing agent used has a major effect on the nucleation process and consequently affects the average size of the particles. This has been reported for MgO nanoparticles which could be produced via CVS technique in the average size ranging from 3, 5 or 11 nm—depending whether N_2_O or O_2_ or dry air were used as the oxidizing agent [122]. Control over particle size can be also realized by varying the reaction temperature [110] since the nucleation and growth kinetics can be controlled by manipulation of temperature and reactant concentration [123]. Reactant delivery, reaction energy input and product separation may also affect the characteristics and quality of the product. These techniques can be modified to obtain desirable attributes in the nanoparticles and eliminate limitations associated with volatility of the reactants and degree of agglomeration. Some examples are laser assisted [124], electrospray assisted [125], thermally activated/pyrolytic, metalorganic, plasma-assisted and photo CVD methodologies [126]. For instance, electrospray assisted chemical vapor deposition (ES-CVD) was employed to synthesize non-agglomerated spherical titanium and zirconium oxide nanoparticles [125]. Djenadic and Winterer [124] have used laser assisted technique to synthesize TiO_2_ and Co-doped ZnO magnetic semiconducting nanoparticles.

##### Combustion method

In this synthesis method, pure metallic precursor is heated by different techniques to evaporate it into a background gas in which the second reactant i.e. oxidizing agent is admixed. The synthesis starts with an initialization in which the metal is only partially heated for the oxidation reaction to start. Thereafter, the heat required for the following metal evaporation is produced in situ by the combustion reactions itself. Even though this process is very successful commercially, the coupling of the particle production to the flame chemistry makes this a complex process that is rather difficult to control. However, the control over partial pressure of oxidizing agent that determines the nucleation and growth can affect the particle size to some extent, as it has been shown for MgO nanosmoke [127]. Nanoparticles of ZnO [128], FeO [129], CuO, Mn_2_O_3_, MgO [127], CdO and Co_3_O_4_ [130] or Ag supported on MgO surface [131], Co_3_O_4_ on CuO nanowire arrays (Co_3_O_4_@CuO) [132], La_0.82_Sr_0.18_MnO_3_ [133]. Another example of using this synthesis route for the production of polymetallic oxides was shown in the work by Vidic et al. [134]. In this paper a phase separation—an existence of both, the hexagonal ZnO and cubic MgO crystal phases—has been demonstrated. Despite this disadvantage relatively good antibacterial efficiency and biocompatibility of ZnMgO nanoparticles were shown. Modifications in combustion technique, such as reported by Lee and Choi who have used a CO_2_ laser to re-heat flame-synthesis technique, affects nanoparticle morphology and degree of agglomeration of TiO_2_ nanoparticles [135]. Wegner et al. [136] have employed a modification by using a critical flow nozzle to extract synthesized titania nanoparticles from the flame to quench particle growth and agglomeration.

##### Template/surface-mediated synthesis

The major strategies employed in this type of fabrication are electrochemical [137], electroless and sol–gel [138], chemical polymerization [139], and chemical vapor deposition [140]. Consequently, as reaction between metal and oxidizing agent may take place in different medium this method can be attributed to both of the previously listed classes of synthesis. The method is based on fabrication of the desired nanomaterial within the pores or channels of a nanoporous template. Depending on the properties of the template, various morphologies of nanomaterial such as rods, fibrils, and tubules, can be prepared. This method can be used to synthesize self-assembly systems with tubular and fibrillary like nanostructures with small diameters [141]. Highly monodisperse nanostructures with enhanced activities, uniform morphology and a high specific surface area can be obtained using this synthesis method [142]. Examples are mesoporous MoO_2_ nanoparticles with improved electrochemical properties [143], α-Fe_3_O_4_ and Co_3_O_4_ [144], Fe_2_O_3_ [145] and mesoporous NiMn_2_O_x_ [144]. The templates used for such synthesis methods mainly are track-etch membranes, porous alumina and other nanoporous structures, like mesoporous zeolites [146, 147]. Carbon nanotubes have been used for the fabrication of a variety of metal oxide nanoparticles like PbO, Bi_2_O_3_, V_2_O_5_, SiO_2_, Al_2_O_3_, MoO_3_, MnO_2_, Co_3_O_4_, ZnO, and WO_3_ [148–150]. The choice of precursor, fixation method and loading allow for the control of nanoparticles size and shape. Sun et al. have established that the size and shape of reaction container along with simple modifications in the container opening accessibility can have significant impact on the crystal growth and thereby the properties such as particle size, mesostructure ordering and crystallinity [145]. In addition, the choice of container has been associated with reproducibility of crystallite size or shape for the same nanomaterials.

#### Biological synthesis

Nature is able to synthesize a variety of metal oxides nanomaterials under ambient conditions [151]. As biocompatibility is one of the most important requirements for any nanomaterial used in the field of nanomedicine, extensive research for synthesis techniques using micro-organisms is currently undertaken. For instance, magnetite nanocrystals have been synthesized in magnetotactic bacteria as a part of their magnetic navigation device [152]. ZnO nanoparticles were synthesized from leaf extracts [153]. Raliya and Tarafdar [154] have synthesized ZnO, MgO and TiO_2_ nanoparticles by using fungus. In these syntheses, an enzymatic reaction replaces the chemicals process which eliminates the production of toxic wastes and is more environment-friendly. In addition, a biological synthesis is lesser energy intensive than its physicochemical counterparts. The particles generated by these processes have higher catalytic reactivity, greater specific surface area if not coated with a lipid layer [155, 156]. In some cases, nanoparticles produced in microorganisms are purified coated with protein corona which confers their physiological solubility and stability. These may be critical for biomedical applications and is the bottleneck of some purification methods. The biological synthesis is supported by the fact that the majority of the bacteria inhabit ambient conditions of varying temperature, pH, and pressure. By varying parameters like microorganism type and strain, its growth phase, culture growth medium, pH, substrate concentrations, temperature, reaction time, addition of non-target ions and a source compound of the wanted nanoparticle it is possible to control size of particle and their monodispersity [157]. Compared to chemical and physical methods, the main drawback associated with biological synthesis is the inability to obtain desired size and/or shape of nanoparticles along with a low yield. Slow in general, this process may take several hours and even a few days. Moreover, the decomposition of formed nanoparticles may take place after a certain period of time. Due to its biocompatibility, however, this process remains very attractive when it comes to the production of potential antibacterial agents.

#### Choice of synthesis method

As presented above, a broad variety of techniques for fabrication of nanostructured metal oxides exists. The reason for it stems mostly from their vast technological applications. Except biological, all described methods can provide metal oxide nanocrystals of high quality, with precisely defined particles size or shape—the properties which play a major role when antibacterial efficiency is under question. However, for most of the above mentioned techniques, it is not possible to establish control over all the involved characteristics simultaneously, more so when synthesizing polymetallic oxide nanoparticles. In this perspective, the most efficient is chemical vapor synthesis that provides in addition a very high crystal purity—similar to other vapor based techniques. Another exceptional advantage of chemical vapor synthesis is, however, the stabilization of otherwise unstable crystal phase. For instance, ZnO in cubic crystal structure can only be obtained under very high pressures. However, CVS allows for c-ZnO to be dispersed within MgO surface [13]. This is very important given that the type of the crystal phase may also affect the antibacterial efficiency of the considered oxide which may exist in more than one structure. However, the relation between crystal phase and antibacterial efficiency is not clearly provided in the literature. For instance, despite a complete phase separation in smoke ZnMgO that occurred in the course of metal combustion synthesis, its surprisingly good antibacterial activity was evidenced [134].

Nanoparticles’ agglomeration, that plays a significant role in determining the antibacterial efficiency, is another issue at hand. The tendency for the agglomeration is favored by electrostatic forces between particles itself, i.e. even when they are not dissolved (Fig. 1). Some of solution-based fabrication techniques use surfactants [42] which, in addition to affecting particles size, tend to decrease the agglomeration degree between particles. In such cases, however, the presence of foreign, mostly organic, groups attached to the surface of primary metal oxide nanoparticles must be considered—the situation where we switch actually to composites and deal no more with pure mono or multi oxides. Moreover, solution based techniques struggle with the problem of contaminations present in a resulting metal oxide product. Indeed, nanoparticles remain frequently contaminated with anions present in the precursor salts despite multiple and obligatory washing cycles.

Another issue that needs simultaneous in-depth study is the cytotoxic nature of these metal oxide nanoparticle. Research on determining the characteristics that can produce concomitant low harmful cytotoxic effects is still in its infancy, especially when polymetallic oxides are considered. Biological method occurs as a good alternative but the studies on biogenic synthesis methods are scanty and much work is necessary to improve their efficiency in a first place. Chemical and physical methods are definitely superior in producing larger quantities of nanoparticles but their main advantage over biological is the ability to control the size and shape. “Biocompatible production” needs, therefore, more active research to widen commercialization prospects.

### Metal oxide nanoparticle in aqueous solution

Physico-chemical properties of metal oxide nanoparticles are surface specific and directly dependent on their surface-to-bulk ratio. Therefore, nanoparticles manipulation and storage may modify their fundamental properties. The classical approach of surface science studies employs experimental techniques which preserve pristine properties of nanoparticles. Such techniques imply ultra-high or at least high vacuum conditions i.e. conditions in which the residual pressure of air components is minimized and the surface modifications negligible. However, biological applications typically expose nanoparticles to aqueous environment in which their surfaces may undergo a series of physico-chemical modifications. Accordingly, nanoparticles characteristics, as well as their dispersion and stability have also to be examined in water and biological media or fluids. Indeed, particle dissolution, aggregation/agglomeration and protein corona formation on the particle surfaces may take place in aqueous solutions leading to properties that strongly differ to the ones characteristic for as-synthesized forms.

#### Stabilization and biocompatibility of metal oxide nanoparticles

Notably, metal oxide nanoparticles dissolute partially in water solutions which leads to the modification of their morphology in which formation of new crystallographic phases may take place [158]. The propensity to dissolute in water depends on the composition and structure of the nanoparticles, as was demonstrated for nano-ZnO [159]. The dissolution rate was also shown to strongly depend on nanoparticles size [160]. The significantly higher dissolution rate was observed for CVS-MgO nanocubes (~5 nm average size) than for smoke-MgO cubes (~80 nm average size) produced by magnesium combustion in air. Small CVS-MgO particles were shown to be completely transformed into Mg(OH)_2_ in a water solution. In contrast, on larger smoke-MgO nanoparticles the formed surface hydroxide led to a self-inhibition resulting in only partial dissolution and surface faceting [160]. In addition, the aggregation of metal oxide nanoparticles in water solutions is a common phenomenon. Among others, a typical example which undergoes strong aggregation in water is TiO_2_ nanopowder [161, 162].

Two strategies for nanoparticle dispersion in water are mainly employed: electrostatic stabilization and steric repulsion. In the course of electrostatic stabilization, particles do not aggregate due to their equal charges i.e. electrostatic repulsion. This method is simple to realize but demands well defined pH and ionic strength of the solution and the control of the presence of reactive species such as OH- or H_3_O^+^ ions that can modify the surface charge of metal oxide particles. Considering steric repulsion, the surface of nanoparticle is modified by an appropriate hydrocarbon polymer or a bio-macromolecule. Such stabilizing molecules can be adsorbed or grafted onto the nanoparticles surface to prevent direct contact between them and, thus, their aggregation. Consequently, the nanoparticles remain dispersed in water solution even upon pH changes or salt concentration [163].

Bovine serum albumin is a commonly used stabilization agent as it spontaneously forms a protein corona around metal oxides particles [162]. The advantage of using albumin lies in its biological role to nonspecifically bind various molecules and its natural and abundant presence in biological fluids, such as blood. As albumin is a charged biomacromolecule, its adsorption on the metal oxides allows both nanoparticle steric and electrostatic stabilization. However, albumin adsorption on nanoparticles is not always stable and may, thus, be inefficient for some applications. For instance, when nano-ZnMgO was added to cell culture medium containing albumin as the most abundant protein, the protein corona consisted of many other proteins from the medium [164]. This indicated that over time the most abundant protein in initially formed corona may be replaced by proteins which are less abundant but have higher affinities to interact with nanoparticles’ surface. In such cases, superior surface-active agents have to be used for effective nanoparticle stabilization in a given medium. For instance, the prevention of nanoparticle aggregation and the achievement of their stable dispersion in an aqueous solution might be obtained by adding a mild detergent, as Tween-20 or P-20. Also, a recent study has shown that nontoxic polycarbonate ethers may efficiently substitute albumin to stabilize TiO_2_ giving a suspension of non-aggregated nano-TiO_2_ in various cell culture media tested [165].

### Antibacterial activity of metal oxide nanoparticles

**Fig. 2:**
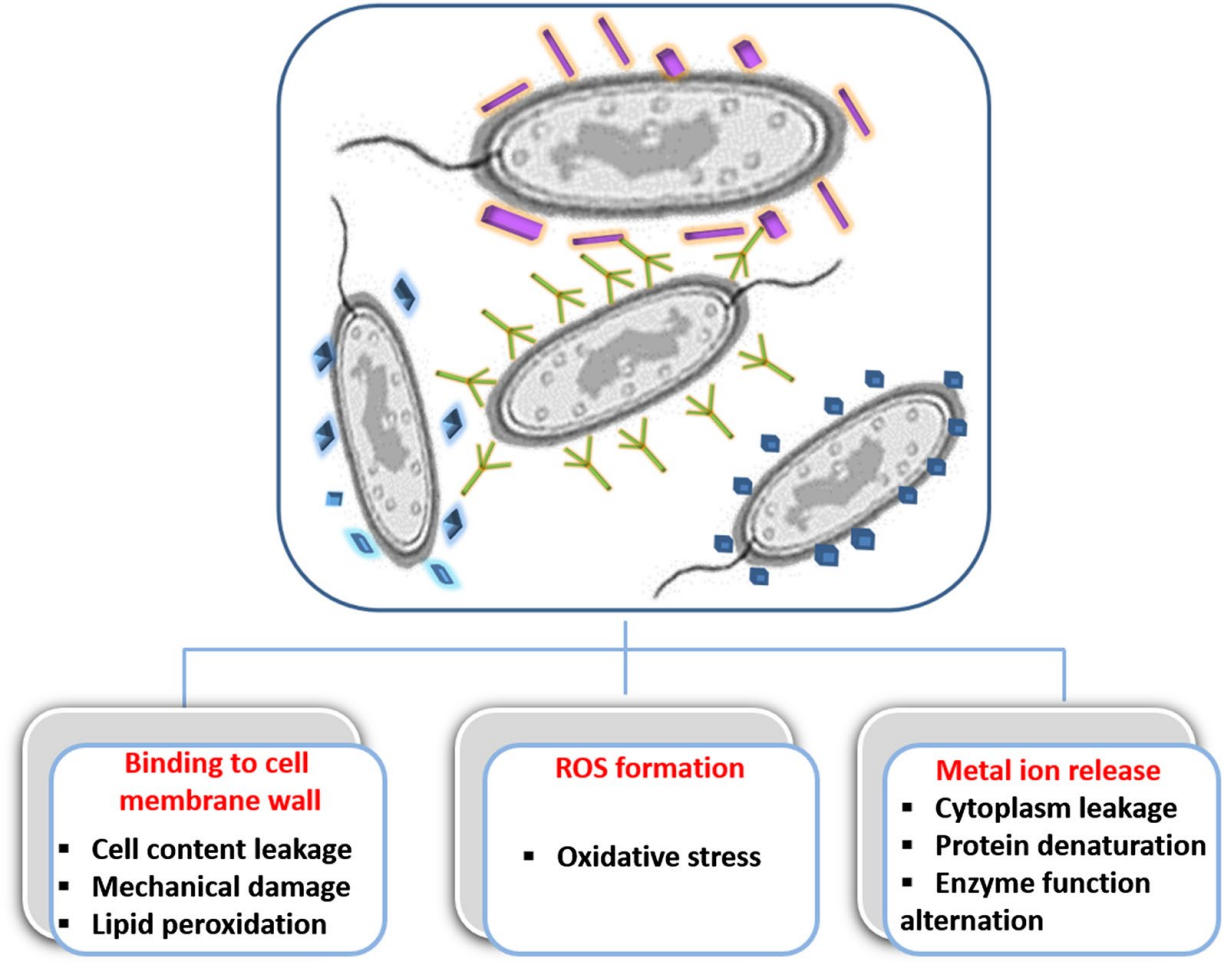
Metal oxide nanoparticles interracting with bacteria. Molecular mechanisms of antibacterial activities of metal oxide nanoparticles

Several metal oxides in form of nanoparticles have been reported to exhibit marked antibacterial activity allowing efficient eradication of various bacterial strains. This fact has attracted significant interest of environmental, agricultural and health care industries that are searching for newer and better agents to control or prevent bacterial infections. Many studies have been undertaken to explain the efficacy and mechanisms of antibacterial action of metal oxide nanoparticles but the existent literature is still controversial and incomplete. It was demonstrated, however, that when applied at well-defined sizes, crystal structure and concentrations, these nanoparticles are highly effective inhibitors against a wide range of bacteria. Although their exact antibacterial mechanism is still under debate, some distinctive mechanisms have been proposed, which include reactive oxygen species (ROS) formation, metal-ion release, particle internalization into bacteria and direct mechanical destruction of bacterial cell wall and/or membrane (Fig. 2). Metal oxide nanoparticles may show bacteriostatic or bactericidal effect. In case of bacteriostatic effect, treated bacteria do not die but stop to reproduce or grow. If treated bacterial cells are removed from the solution containing nanoparticles, they re-start to grow. This can be easily tested by plating these bacterial cells onto new nanoparticle-free agar. In case of bactericidal effect, no bacterial colonies can be observed upon re-plating treated bacteria onto nanoparticle-free agar. Depending on the experimental conditions, nanoparticle concentration and bacterial strain, a particular type of metal oxides nanoparticle may have bacteriostatic or bactericidal effect as shown for ZnO [166] or TiO_2_ [167].

Different ions, small molecules (such as H_2_O_2_), free radicals (like, OH, ^1^O_2_) or superoxide ions (such as ${\text{O}}_{2}^{ - }$) are examples of highly reactive ROS species which can be produced on the surface of metal oxide nanoparticles and can induce bacterial cell death. ROS-induced damages and bacterial death comprise oxidative stress, oxidative lesions and membrane lipid peroxidation. In addition, ROS can harm bacterial components such as proteins and nucleic acids. For instance, oxidative stress induced by Ag_2_O nanoparticles was shown to damage the DNA of *E. coli* which led to the interruption of the bacterial cell cycle and induction of bacterial death [168]. Also, CuO nanoparticles were shown to generate ROS, namely superoxide anions, when adsorbed onto the bacterial cell surfaces or internalized into bacterial cells. Formed ROS induced bactericidal effect in both Gram-positive (*S. aureus*) and Gram-negative (*E. coli*) bacteria [169].

The physicochemical characteristics of metal oxide nanoparticles, such as size, crystal structure defects, composition and surface charge, are directly associated with enhanced antibacterial effects. The synthesis and treatment procedures employed can tune these characteristics, as discussed in the previous sections, and hence produce the desired antibacterial efficacy. For instance, nanoparticles of smaller sizes (<20 nm) can easily penetrate into bacterial cells and may release toxic metal ions upon dissolution [170]. Thus, smaller particles are usually the most efficient antibacterial agents. However, this is not the case when decrease in size leads to enhanced aggregation. Also, defects present at the nanoparticles’ surface influence strongly antibacterial efficiency. Point defects, such as atoms at edges and in corners give rise to an abrasive surface that may cause the injury of the bacterial cell wall or membrane. For instance, it was proposed that partial dissolution of nano-ZnO in water medium results in formation of surface defects giving an uneven surface texture due to rough edges and corners. This surface roughness was shown to be responsible for mechanical damage of the cell membrane of *E. coli.* Wang et al. [171] have also suggested that the crystallographic orientation and type of surface plane can influence antibacterial efficiency of ZnO nanowires. They showed that randomly oriented ZnO nanowires were more efficient in killing *E. coli* than regularly oriented ones. This is probably due to different spatial arrangements of ZnO.

Surface charge was also shown to play an important role in membrane damage and particle internalization. Bacterial membranes and cell walls are typically of negative total charge. Electrostatic attractions can occur between bacterial surfaces and metal oxide nanoparticles of positive zeta-potential, like observed for positively charged nano-ZnO and negatively charged *C. jejuni* cells. Xie et al. [172] proposed that upon binding to bacterial surface, ZnO nanoparticles disrupted the cell membrane causing morphological changes and measurable membrane leakage in *C. jejuni*. Moreover, even particles of negative zeta potential may damage cell membranes since interactions cannot only be electrostatic, but Van der Waals and hydrophobic as well. Metal oxide nanoparticles may specifically bind some moieties within membrane barrier surface such as phosphate, amine or carboxyl groups in lipids and proteins and subsequently induce bacterial death. It is worth noting that metal oxide nanoparticles remain tightly bound to the surface of damaged or dead bacteria which may modify their effective concentration in the given solution over time.

Since metal oxide nanoparticles with varying physicochemical characteristics exhibit different antibacterial mechanisms and effects, oxide nanoparticles with a combination of two or more metals can be developed for efficient elimination of various bacterial strains including those highly resistant to existing treatments. Table 1 summarizes some examples of multi-metal oxide nanoparticles tested for their applications in eradication of different bacterial strains. Interestingly, some multi-metal oxide nanoparticles show higher antimicrobial activity when compared to their pure components of similar size.

**Table 1:** Some examples of mixed and doped metal oxide nanoparticles that were tested for their antibacterial activity

Metal oxides nanoparticle	Synthesis/doping method	Bacterial strain tested	References
Zn/Fe oxide	Sol gel	*S. aureus; E. coli*	[173]
Zn/Mg oxide	Combustion	*E. coli; B. subtilis*	[134]
ZnO/Au	Photo-reduction	*E. coli; S. aureus*	[174]
TiO_2_/Ag	Reactive magnetron sputtering	*S. aureus*	[175]
Fe_3_O_4_/Ag	Template based	*S. aureus*	[176]
Ta-doped ZnO	Sol gel	*B. subtilis; S. aureus; E. col; P. aeruginosa*	[177]
Fe-doped ZnO	Sol gel	*E. coli*	[178]
Ce-doped ZnO	Sonochemical	*E. coli*	[179]
Nd-doped ZnO	Co-precipitation	*E. coli; K. pneumoniae*	[180]
Zn-doped CuO	Sonochemical	*E. coli; S. aureus*	[181]
Zn-doped TiO_2_	Electrospinning	*E. coli; S. aureus*	[182]
Ag-doped TiO_2_	TiO_2_-Sol gel Ag-doped TiO_2-_Solvothermal	*E. coli; S. aureus*	[183]
Cu-doped TiO_2_	Flame Synthesis	*M. smegmatis; S. oneidensis*	[184]
Cu-doped TiO_2_	Co-precipitation	*E. coli*	[185]
Li-doped MgO	Sol gel	*E. coli*	[186]
Cu-doped MgO	Co-precipitation	*E. coli*	[187]
Ag-doped SiO_2_	SiO_2-_Sol gel Doped SiO_2_- Co-precipitation	*P. aeruginosa; S. aureus; E. coli*	[188]
Mn- and Fe-doped ZnO	Co-precipitation	*S. aureus; E. coli; K. pneumoniae; S. typhi; P. aeruginosa; B. subtilis*	[189]
Zn- and/or Y-doped TiO_2_	Sol gel	*C. albicans; S. aureus*	[190]
${\text{Zn/Ce/SO}}_{4}^{2 - }$-doped TiO_2_	Sol gel	*E. coli; S.aureus*	[191]
Ag-TiO_2_-doped SiO_2_	Sol gel	*E. coli*	[192]

**Fig. 3:**
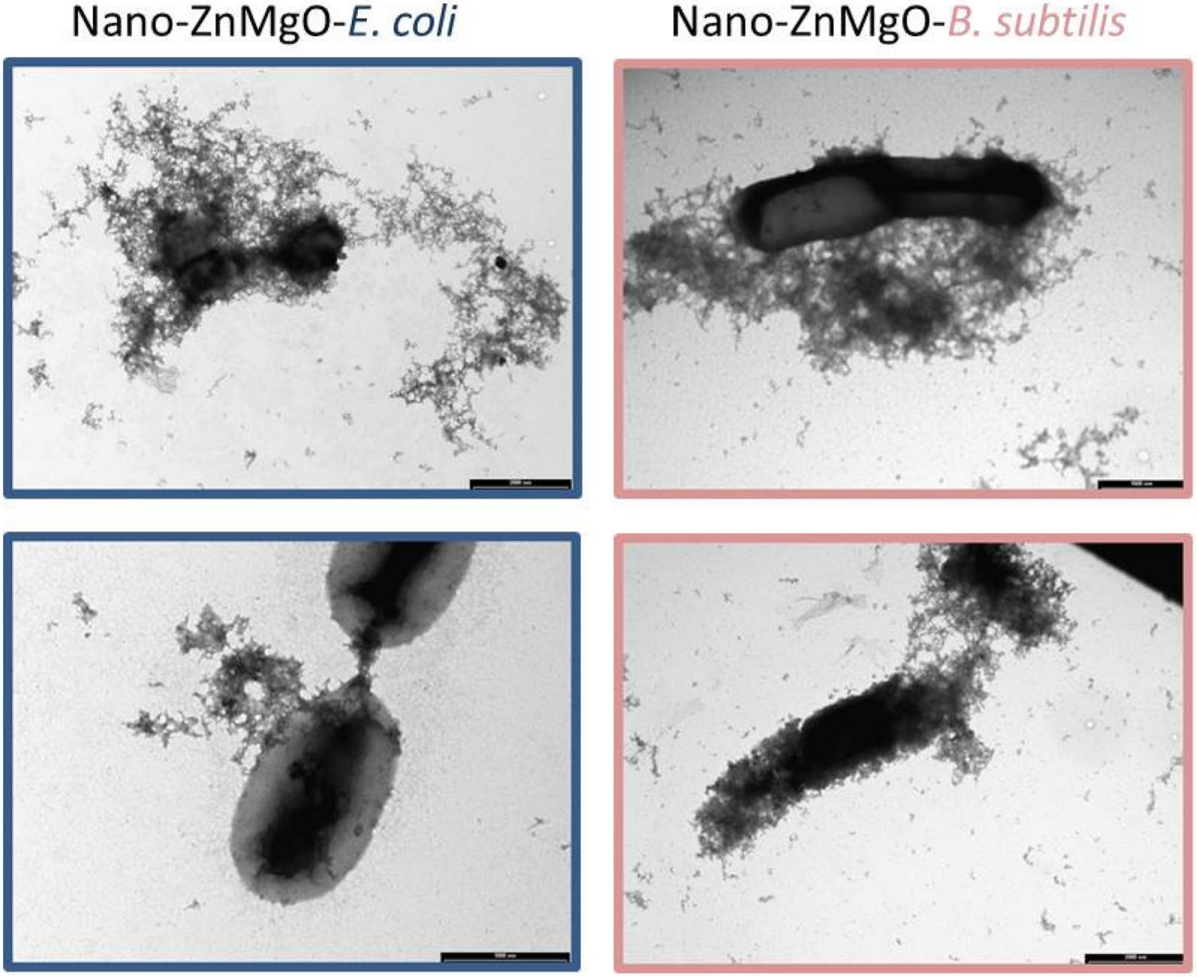
Treating* E. coli* and* B. subtilis* with ZnMgO nanoparticles. TEM images of Gram-negative bacteria *E. coli* (*blue*) and Gram-positive bacteria *B. subtilis* (*rose*) treated with mixed ZnMgO nanoparticles. Note nanoparticles association with bacterial cells, the leakage of cell content and particles aggregations in the bacterial grown medium. Images were obtained at MIMA2 MET platform in INRA Jouy en Josas. *Bar* 1000 nm

For instance, nanostructured ZnMgO produced by combustion technique exhibit advantageous properties from both of its pure components: high antibacterial activity of nano-ZnO and low cytotoxicity of nano-MgO [134]. This mixed metal oxide inhibited Gram-positive bacteria (*B. subtils*) completely and Gram-negative bacteria (*E. coli*) partially upon 24 h treatment [134]. ZnMgO nanoparticles were shown to damage bacterial cells by causing extensive injury to membranes that resulted in a leakage of the cell content as illustrated in Fig. 3. Comparatively, pure ZnO nanorods and nanotetrapods exhibited the highest but nonselective activity as they completely eradicated both bacterial strains and mammalian HeLa cells, under the same treatment protocol [134]. In contrast, pure MgO nanocubes only partially inhibited bacterial growth being at the same time harmless to mammalian cells.

In case of Zn/Fe oxide nanocomposites, antibacterial effectiveness similar to that of ZnO nanoparticles, was observed [173]. However, no particle agglomeration, typical for nano-ZnO in water solutions was detected. Compared to nano-ZnO, the pure Fe_3_O_4_ lacks significant antibacterial efficiency, but exhibits good colloidal stability [173]. It was observed that both the antibacterial effect and stability of Zn/Fe oxide nanocomposite in an aqueous medium can be optimized by changing the ratio of Zn/Fe. The study suggested that hydroxyl radicals were formed at the surface of zinc oxide. These active oxygen derivatives were proposed to damage bacterial cells of *E. coli* and *S. aureus*. Since similar mechanisms were not observed for zinc ferrite, it appears that iron oxide contributes only towards good colloidal stability of the composite. In another study, Fe^3+^-ions were used to dope nano-ZnO in order to induce the formation of lattice defects in ZnO nanocrystals and thus to enhance its antibacterial efficiency. It was shown that Fe-doped ZnO nanoparticles efficiently inhibited *E. coli* bacterial growth without being toxic to mammalian cells [193]. Fe^3+^-ions acted as an impurity in the ZnO nanostructure that enhanced the overall antimicrobial activity. Similarly, it was observed that sea urchin-like ZnO doped with 5 % iron had a strong antimicrobial activity, as it killed up to 95 % *C. albicans* and *A. flavus* [194]. Inserting Fe^3+^-ions into ZnO lattice increased antibacterial efficiency by decreasing the size of ZnO nanoparticles and favoring the formation of sea urchin-like structure. Moreover, Fe^3+^-ions have the capacity to kill bacteria by destroying both cell walls and membranes due to their strong reduction ability. Also, binding Fe^3+^-ions to biomolecules may cause protein denaturation, DNA damaging and enzyme function alternating.

Guo et al. [177] have reported that when ZnO nanoparticles were doped with Ta, the bactericidal activity was revealed to be higher than that of pure nano-ZnO. The introduction of Ta^5+^-ions into ZnO crystal structure induced changes in structure, morphology and surface defect concentration giving larger lattice parameter, smaller grain size and more active defect sites and hydroxyl groups—formed upon particles dissolution in water. In consequence, the surface reactivity of nano-ZnO could be dramatically increased by Ta-doping. The antimicrobial activity of Ta-doped ZnO nanoparticles was tested on *B. subtilis*, *S. aureus* (Gram-positive bacteria) and *E. coli* and *P. aeruginosa* (Gram-negative bacteria) under dark ambient and visible light irradiation. The incorporation of Ta^5+^-ions into ZnO significantly improved the bacteriostatic effect of ZnO nanoparticles on *E. coli*, *S. aureus* and *B. subtilis* in the absence of light, while both Ta-doped ZnO and pure ZnO nanoparticles showed increased bactericidal efficacy on *P. aeruginosa*, *E. coli* and *S. aureus* under visible light illumination. It was proposed that the high valence of Ta^5+^ might generate Zn vacancy or oxygen interstitial to keep the electric neutral equilibrium in the crystal structure. This, in return, increased the productions of ROS. In addition, the high valence of Ta^5+^ increased electrostatic attractions between metal oxide nanoparticles and the bacterial surface which also facilitated the bactericidal action. The differences in bactericidal efficiencies observed with various strains may originate from different structure and composition of the bacteria tested. For instance, Gram-negative bacteria possess a double membrane bilayer while Gram-positive bacteria are limited only by one lipid bilayer.

In another study, He et al. [174] have observed that deposition of small Au particles of 3 nm diameter onto the surface of ZnO nanoparticles—at a very low ZnO/Au molar ratio (0.2 %)—significantly enhanced the photocatalytic and antibacterial activity of ZnO. Indeed, deposition of Au onto ZnO nanoparticles resulted in production of holes and electrons at the particle surface which dramatically increased light-induced generation of hydroxyl radical, superoxide and singlet oxygen. When incubated with *E. coli*, the ZnO/Au hybrid nanostructures showed about three times higher antibacterial efficiency than pure ZnO nanoparticles. Also, ZnO nanoparticles doped with both Mn and Fe ions (10 % molar ratio) exhibited higher antibacterial activities as compared to 1 % loading or pure ZnO when incubated with *S. aureus*, *E. coli*, *K. pneumoniae*, *S. typhi*, *P. aeruginosa* and *B. subtilis* [189]. The enhancement in antimicrobial effectiveness was attributed to the increased generation of ROS due to the synergistic effects of Mn and Fe loading. When bound to the bacterial surface Fe- and Mn-doped ZnO nanoparticles induced an apparition of holes on the membrane surfaces, which subsequently led to cell death. Interestingly, these doped nanoparticles shown higher efficiency against Gram-negative than against Gram-positive bacteria.

Similarly, TiO_2_ nanoparticles coated with Ag nanoparticles showed increased antibacterial effectiveness against *S. aureus* compared to pure TiO_2_ [175]. The proposed mechanism involved a direct mechanical destruction of bacterial cells upon binding of nanoparticles to their surfaces. The final effect was enhanced by bactericidal activity of released silver from the particle surface. In addition to enhanced antibacterial activity, Ag/TiO_2_ hybrid structures showed higher durability compared to pure TiO_2_. In another study, TiO_2_ nanoparticles were doped with zinc and/or yttrium in order to increase their antibacterial activity [190]. It was shown that bactericidal efficiency of the obtained Zn–Y/TiO_2_ nanomaterials strongly depended on the synthesis procedure but also on composition and irradiation with visible light. Zinc-doped TiO_2_ nanoparticles were much more efficient than yttrium-doped ones when *C. albicans* or *S. aureus* were treated for 30 min upon visible light irradiation. However, the double-doped Zn–Y/TiO_2_ nanoparticles revealed the highest antibacterial activities compared to pure TiO_2_, Zn-doped TiO_2_ or Y-doped TiO_2_ when exposed to visible light. Since antibacterial activity of Zn–Y/TiO_2_ nanoparticles was weaker in dark than that in visible irradiation the mechanism seemed to be related to the generation of toxic hydroxyl radical upon illumination. Moreover, co-doped nanoparticles were shown to release Zn and Y ions, both highly toxic for bacteria since they easily penetrate cell membrane barrier.

Another approach consisted in applying a mild solvothermal method to synthesize Ag-doped TiO_2_ nanosheet films. When film attachment method was used to estimate Ag–TiO_2_ activities against *E. coli* and *S. aureus* growth, the excellent performance in killing bacteria under UV light and in the dark was observed [183]. Ag-doped TiO_2_ combined the advantages of highly efficient antibacterial effects of Ag with low cost production of TiO_2_ nanoparticles. In addition to acting as an antimicrobial auxiliary agent in this complex material, silver also acted as a sink for electrons and redox catalyst which enhanced the photo-oxidation ability of TiO_2_. Indeed, Ti^4+^-ions in TiO_2_ substituted by monovalent Ag^+^-ions increased the density of defects and generation of oxygen vacancies, which improved antibacterial performance of the nanosheets. However, possible applications of this nanomaterial are limited by the cytotoxicity of Ag–TiO_2_ nanocomposites, especially those that contain more than 4 % of silver. Likewise, Cu-doped TiO_2_ have been observed to exhibit higher antibacterial activity than pure nano-TiO_2_. Moreover, Cu-doped TiO_2_ nanoparticles of 20 nm diameter synthesized by a flame aerosol method significantly reduced the growth rate of *M. smegmatis*, but did not affect the growth of *S. oneidensis* at 20 mg/L. In contrast, pure TiO_2_ had no effect on growth of the two strains even at 100 mg/L [184]. Cu-doped TiO_2_ nanoparticles, similarly to non-doped TiO_2_, agglomerated in the bacterial medium and, thus, probably did not directly damage bacterial cellular structures. The overall inhibitory effect on *M. smegmatis* growth suggested that Cu^2+^ and TiO_2_ might have synergistic effects and that TiO_2_ nanoparticles served as a carrier and concentrator of highly efficient copper ions which resulted in an enhancement of antibacterial efficiency compared to pure CuO. The toxicity of Cu-doped TiO_2_ was probably driven by the release of Cu^2+^-ions since the corresponding antibacterial effectiveness increased with an increase of copper content. Interestingly, *S. oneidensis MR*-*1* was able to tolerate high concentrations of Cu^2+^-ions as capable to enzymatically reduced ionic copper in a culture medium.

Pure nano-MgO exhibits only mild antimicrobial activity against both Gram-positive and Gram-negative bacteria but has an advantage in being synthesized from available and economical precursors. Metal-ion doping has been shown to be an effective method to improve its antibacterial efficiency. However, Rao et al. [195] have shown that doping MgO with different metal ions may give opposite effects on nanoparticles’ antibacterial properties. Li-doped MgO was more efficient than pure MgO, while Zn- and Ti-doped nano-MgO displayed poorer antibacterial activity than MgO. The authors concluded that doping with Li^+^ promoted the generation of oxygen vacancies and increased the basicity of the oxide, which favorited generation and stabilization of superoxide anion, ${\text{O}}_{2}^{ - }$. In contrast, Ti^2+^ and Zn^2+^, having higher valence than Li^+^, less efficiently favored these two phenomena although Ti-doped MgO was somehow more efficient than Zn-doped MgO in eliminating *E. coli*—which was ascribed to smaller sizes of Ti-doped MgO compared to those of Zn-doped MgO nanoparticles.

Although CuO nanoparticles have been shown to possess marked antibacterial activities, they usually have to be applied in higher doses and are not efficient against all bacterial strains. However, when used in a nanocomposite form, CuO has been shown to be highly efficient. For instance, Zn-doped CuO nanocomposite in a colloidal suspension form or deposited on the fabric shown a 10,000 times enhancement in the antibacterial activity against *E. coli* and *S. aureus* bacteria compared to pure-ZnO or CuO [181]. Physicochemical characterization of the nanocomposite suggested that Zn-ions were incorporated into the crystalline lattice of CuO. Such nanocomposite produced larger amount of ROS in an aqueous solution and subsequently more toxic OH radical, superoxide anions and singlet oxygen than its pure metal oxides components. Although it is difficult to compare antibacterial efficiency of one nanocomposite towards different bacterial strains, it appears that bacteria rich in amine and carboxyl groups at the surface, like *B. subtilis*, bind more strongly CuO and thus is more sensitive to its bactericidal effects.

### Toxicity of mixed metal oxide nanoparticles

**Fig. 4:**
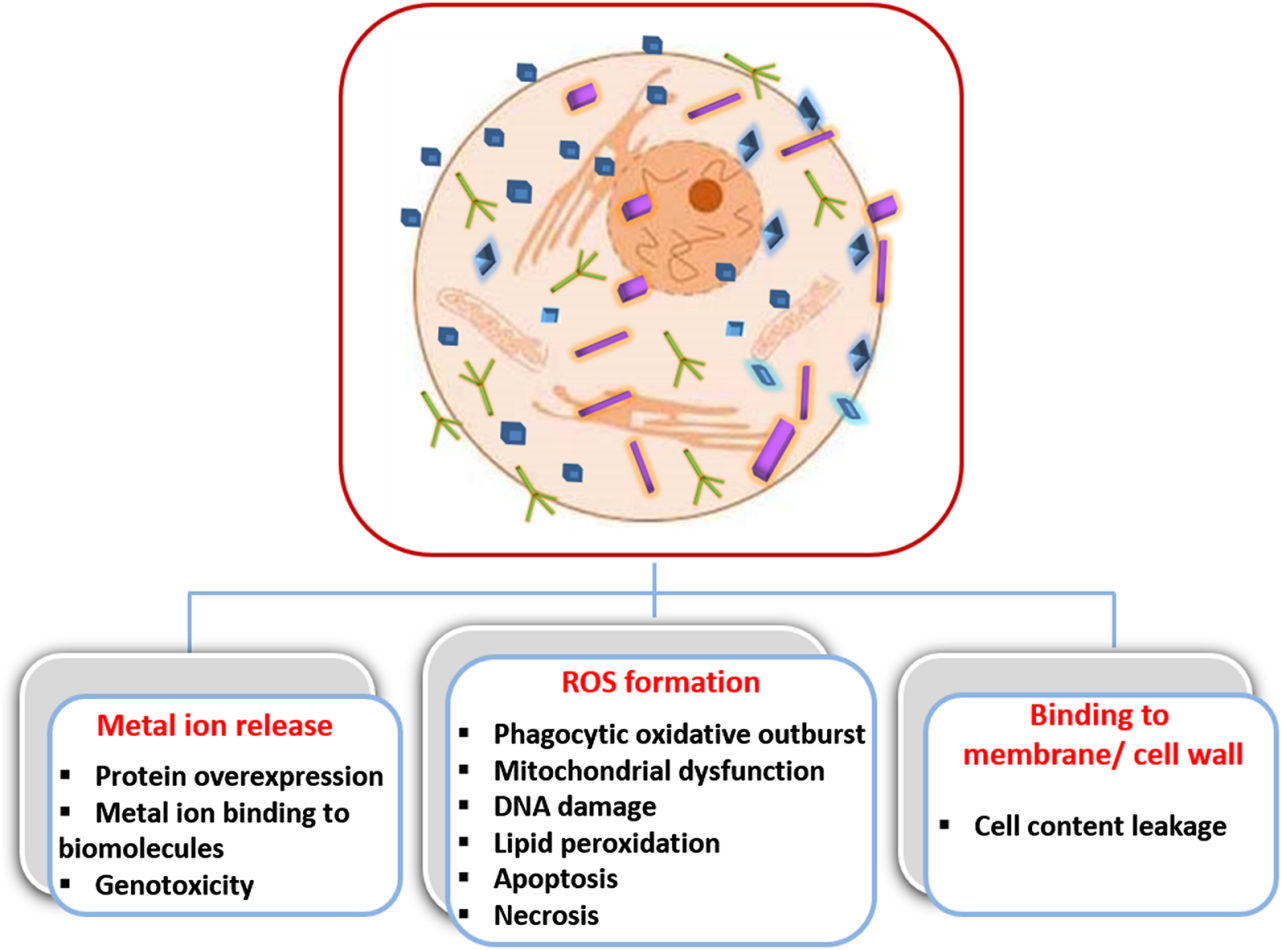
Metal oxide nanoparticles interracting with mamallian cell. Various mechanisms of metal oxide nanoparticles toxicity towards mammalian cells

Understanding the mechanisms involved in interaction of metal oxide nanoparticles with mammalian cells is required for any safe practical application. Presently, we lack knowledge about the general mechanism by which these nanoparticles bind and interact with eukaryotic cells. Similar to antibacterial activity, the cytotoxicity of metal oxide nanoparticles is also dependent on their physicochemical characteristics. Metal oxide nanoparticles of similar size but of different compositions usually show varying cytotoxic effects. For instance, when toxicities of CuO, TiO_2_, ZnO, CuZnFe_2_O_4_, Fe_3_O_4_ and Fe_2_O_3_ were compared in vitro using human alveolar epithelial cells A549, CuO was shown to induce a high percentage of cell death together with DNA damage, while TiO_2_ could only trigger DNA damage [196]. Interestingly, pure Fe_3_O_4_ and Fe_2_O_3_ were almost harmless while mixed CuZnFe_2_O_4_ nanoparticles strongly damaged DNA. Such results strongly suggest that metal oxide nanoparticles of different compositions interact with living cells through different mechanisms—some of which are schematically presented in Fig. 4.

Lai et al. [197] have shown that ZnO nanoparticles are the most, TiO_2_ nanoparticles the second most and MgO nanoparticles the least effective in induction of human cell death. A study of 19 different metal oxide nanoparticles suggested that the most important factor that determines their toxicity is the inherent toxicity of the metal-ions released [198]. Another recent study of 11 types of metal oxide nanoparticles of similar sizes (<20 nm diameter) suggested that differences in their toxicities might be explained by two principal aspects: release of metal-ions, which is observed as the main mechanism for ZnO and CuO, and induction of ROS generation, observed for Mn_3_O_4_ and Co_3_O_4_ [199]. Interestingly, all nanoparticles tested in this study were shown to be internalized by A549 cells. This suggests that ROS formation and metal-ion release may be triggered from internalized nanoparticles within cells.

Presently, surface coating of metal oxide nanoparticles is employed to modify their toxicity. Nevertheless, the same coating may enhance or reduce the toxic effects depending on their initial surface properties. This was shown for different crystals of nano-ZnO stabilized with trichlorododecylsilane [200]. Probably, the final toxicity reflects physicochemical modifications obtained upon coating such as changes in particle aggregation state, dissolution, zeta potential, and ion and free radical releasing to a solution. Thus, the primary determinant of particle toxicity seems to be its starting surface property and not the coating. Furthermore, the same nanomaterial may show different reactivity, and consequently, toxicity in different media. For instance, the toxicity of nano-ZnO comes partially from the released Zn^2+^-ions into aqueous biological media. It was shown that its toxicity can be lowered using a medium containing phosphate ions [201]. Indeed, the formation of Zn-phosphate inactivates the hazardous Zn^2+^-ions. Similarly a metal-ion chelator diethylene triamine pentaacetic acid can be used to decrease toxicity of metal oxide nanoparticles [202]. Although mild detergents used preventing nanoparticle aggregation are supposed to be interactive, they may additionally alter the toxicity of a given nanoparticle [203].

Finally, the toxicity can also be reduced by doping metal oxide nanoparticles with other metal ions. For instance, nano-ZnO released toxic Zn^2+^-ions and generated ROS causing mitochondria perturbations, cell inflammation and induced cytotoxicity in treated lungs and embryos [204]. All these pro-oxidative and pro-inflammatory effects were reduced by iron doping of nano-ZnO [204]. A uniform distribution of Fe atoms throughout the ZnO crystal structure enhanced the crystal stability in an aqueous solution and reduced dissolution of doped nanoparticles in biological media [205]. Nano-ZnO was more effective in inducing cellular death than nano-MgO [134] but, surprisingly, mixed nano-ZnMgO nanoparticles, containing less than 5 % of zinc were inoffensive to mammalian cells, thus, behaving safely as a pure nano-MgO [134].

Penetration of metal oxide nanoparticles in eukaryotic cells may be prevented by particles binding to specific biomolecules, such as membrane proteins [5]. In other cases, internalized nanoparticles are degraded in cellular endosomes or liposomes and then metabolized. Consequently, such metal oxide nanoparticles are considered safe as they neither affect cell viability nor induce apoptosis. The specific features of nanoparticle interaction with cells depend on the surface energy of the particles, which may be modulated by synthesis procedure or functionalization of their surfaces. The practical application of metal oxide nanoparticles as bactericidal agents is, thus, possible at certain conditions and particle concentrations at which there is low or no toxicity against mammalian cells, as demonstrated for ZnO [5], Fe_2_O_3_ [206] or Ag_2_O_3_ [207].

The application of metal oxide nanoparticles as new antibacterial agents strikingly depends on their cytotoxic nature. Meanwhile, it is important to mark that many studies dealing with cytotoxicity of metal oxide nanoparticles are being done with nanoparticles of not well characterized physicochemical properties. Also, the standardized testing procedure for toxicity assessment of nanoparticles is lacking. This implies that our understanding of cytotoxic mechanisms is incomplete and non-uniform. Taking into account that metal oxide nanoparticles are a class of nanomaterials with the highest global annual production, we expect that the progress in addressing their cytotoxicity will be made rapidly.

## Conclusions

Multi-metal oxide nanoparticles are promising candidates for antibacterial applications if the synergic effects of their constituents are effectively harnessed. Numerous already existing synthesis methods provide a rich base that may fuel research devoted to such applications. This is particularly important keeping in mind that the type of the synthesis affects properties like size, shape, morphology, dispersity, presence and type of stress and defects in the crystal which in turn determines their interaction with bacterial and mammalian cells. The reactivity may also be determined by their solubility and degree of agglomeration. Even though no general conclusion has been established regarding the mechanism of metal oxide nanomaterials interacting with living cells and microorganisms, the commonly proposed once are: ROS formation, interaction with cell membrane, particle internalization and binding with specific targets such as proteins or DNA. In polymetallic oxides, physicochemical parameters of the corresponding components are altered which may lead to novel reactivity towards living organisms. Some multi-metal oxide nanoparticles have shown lesser tendency to aggregate in biological solutions and fluids resulting in an increased antibacterial activity while being highly biocompatible when compared to their components. Taking into account the numerous pure metal/metal oxide components that can be combined to obtain desired complementary effects a plethora of polymetallic oxide nanoparticles can be created with properties specific in terms of their antibacterial activity, biocompatibility and monodispersity in biological media.

## Abbreviations

ROS: reactive oxygen species
CVD: chemical vapour deposition
CVS: chemical vapour synthesis
ES-CVD: electro-spray assisted chemical vapor deposition
